# Nanosilver as a novel biocide for control of senescence in garden cosmos

**DOI:** 10.1038/s41598-020-67098-z

**Published:** 2020-06-24

**Authors:** Ewa Skutnik, Agata Jędrzejuk, Julita Rabiza-Świder, Julia Rochala-Wojciechowska, Monika Latkowska, Aleksandra Łukaszewska

**Affiliations:** Section of Ornamental Plants, Institute of Horticultural Sciences, Warsaw University of Life Sciences, Nowoursynowska 166, 02-787 Warsaw, Poland

**Keywords:** Physiology, Plant sciences

## Abstract

To prolong their vase life, cut flowers are commonly kept in holding solutions. These must include a biocide to retard bacterial growth. In this study, the effect of nanosilver (NS) on certain aspects of senescence in cut garden cosmos (*Cosmos bipinnatus*) flowers was compared to that of the commonly used 8-hydroxyquinoline citrate (8-HQC). In combination with sucrose, both biocides prolonged cosmos vase life but did not prevent the occurrence of stem blockages. NS was more effective in limiting a reduction in endogenous soluble carbohydrates. The malondialdehyde (MDA) contents increased in senescing ray florets, both in intact and control cut flowers held in water. Both biocides were comparably effective in limiting this effect. The hydrogen peroxide content tripled in intact flowers but dropped in flowers held in water or the 8-HQC solutions; in flowers kept in NS solutions its increase was moderate. Also, the catalase activity increased in intact flowers but dropped in all cut flowers. Both biocides had similar effects on the enzyme activity, in both pure solutions and with sucrose. Most of these parameters were not significantly correlated with vase life. Overall, the effect of nanosilver on senescence in cut cosmos flowers was similar to that of 8-HQC.

## Introduction

Growing consumer interest in new cut flower species drives field production of seasonal, locally grown flowers^1^. Garden cosmos – also known as lace cosmos because of its delicate pinnate foliage - is gaining popularity not only as a garden or a bedding plant but also as cut flowers. Diversity of forms and colors make the species popular among florists and customers but its postharvest longevity is short, usually 4–6 days^1^. However, this longevity can be doubled by making use of the so-called flower food, i.e. holding solutions containing a biocide and a sugar^2^. These two components are crucial in extending the vase life as they delay flower senescence. Senescence usually occurs faster in cut flowers than in those left on mother plants. This was clearly illustrated by de Stigter^3^ on cut and intact hydroponically grown ‘Sonia’ roses observed under the same environmental conditions and confirmed by subsequent studies on rose^4^, lilac^5^, clematis^6^ and peony^7^.

The processes of senescence are accelerated mainly by water stress caused by improper water balance in cut stems. Such stems, even when held in water, are unable to absorb sufficient amounts of water to compensate for the transpiration losses. Vessels in cut stems become obstructed by blockages of various nature, with the presence of microbes in vase water being the most common^8^. For this reason, biocides in holding solutions are crucial for the postharvest longevity of cut flowers. For over a half a century the esters of 8-hydroxyquinoline have been used as potent biocides, in mixtures with sucrose. This is called the standard preservative^9^. Nanosilver [NS] has emerged as a new biocide for cut flowers after the use of toxic silver nitrate had been outlawed^10^. Though it was enthusiastically accepted in postharvest physiology recent publications warn against its environmental toxicity^11–13^. While the effects of NS on cut flowers have been demonstrated in several species^14–18^ its mode of antimicrobial action is not yet fully understood. This justifies tests on the role of NS in controlling senescence-related processes, especially in comparison with recognized biocides.

After harvest, endogenous carbohydrates sustain respiration that provides energy for the continuation of life. When these sugars become depleted, cut flowers can use those provided in a preservative, usually sucrose. Sucrose is taken up from the holding solution and metabolized, serving not only as a respiratory substrate but also increasing the *osmoticum*, thus improving the water balance and petal turgidity. The latter positively affects cell membranes which normally lose their properties during senescence, due to the lipid peroxidation. These phenomena can be monitored by measurements of the relative water content (RWC), the levels of the malondialdehyde (MDA), or of such cell sap parameters as the pH or electric conductivity (EC). All of these changes due to decreasing semi-permeability of the cell membranes. In cut cosmos flowers, sugar-containing preservatives reduced the electric conductivity of the cell sap relative to water control^19^.

One of the phenomena related to senescence is oxidative stress. This occurs when the production of the reactive oxygen species (ROS) prevails over the plant’s ability to scavenge them. ROS are produced in every living cell and are the side products of oxygen metabolism. When their generation rate exceeds that of the cell’s defense ability, an oxidative stress occurs^20^. One such ROS is hydrogen peroxide which participates in many developmental events but in high concentrations it is damaging to most organelles, including cell membranes^20^. Increasing amounts of hydrogen peroxide were observed in numerous cases, including senescing gladiolus flowers, peaking at the end of the flower life^21^.

Catalase (CAT, EC 1.11.1.6) is an important enzyme of the oxidoreductase group which protects cells against H_2_O_2_ and other forms of ROS^22^. According to Chakrabarty *et al*.^23^ the catalase activity in cut chrysanthemum flowers kept increasing until full bloom and then dramatically dropped. As there are relatively few reports on the effects of preservatives on the oxidative stress in cut flowers this aspects has been included in the current study. The overall goal of this study was to compare the effects of NS to those of the routinely used 8-HQC on the longevity of cut cosmos flowers, formation of blockages in stem vessels and on some aspects of senescence occurring in ray florets, such as changes in the relative water content, total glucose, MDA and H_2_O_2_ levels and the catalase activity. The latter aspects were also compared in cut flowers and flower heads left on the mother plants.

## Results

The effects of NS and 8-HQC, both in pure solutions and with 2% (w/v) sucrose, on the vase life in garden cosmos are listed in Table 1. In pure solutions neither of the biocides had any effect on the longevity of cut flowers. However, when present in solutions of 2% (w/v) sucrose both produced comparable increases in the vase life, by 53–69% relative to water control.

**Table 1:** The effect of two biocides on the vase life of cut flowers of garden cosmos.

Treatment	Vase life (days)
H_2_O (control)	3.6 ± 1.3 a^a^
8-HQC	3.4 ± 0.5 a
8-HQC + 2% (w/v) S	6.1 ± 1.4 b
NS	4.1 ± 0.4 a
NS + 2% (w/v) S	5.5 ± 0.8 b

^a^Means followed by the same letter do not differ significantly at α = 0.05. Values are expressed as the means ± SD. 8-HQC – 8-hydroxyquinoline citrate; S – sucrose; NS – nanosilver.

**Figure 1:**
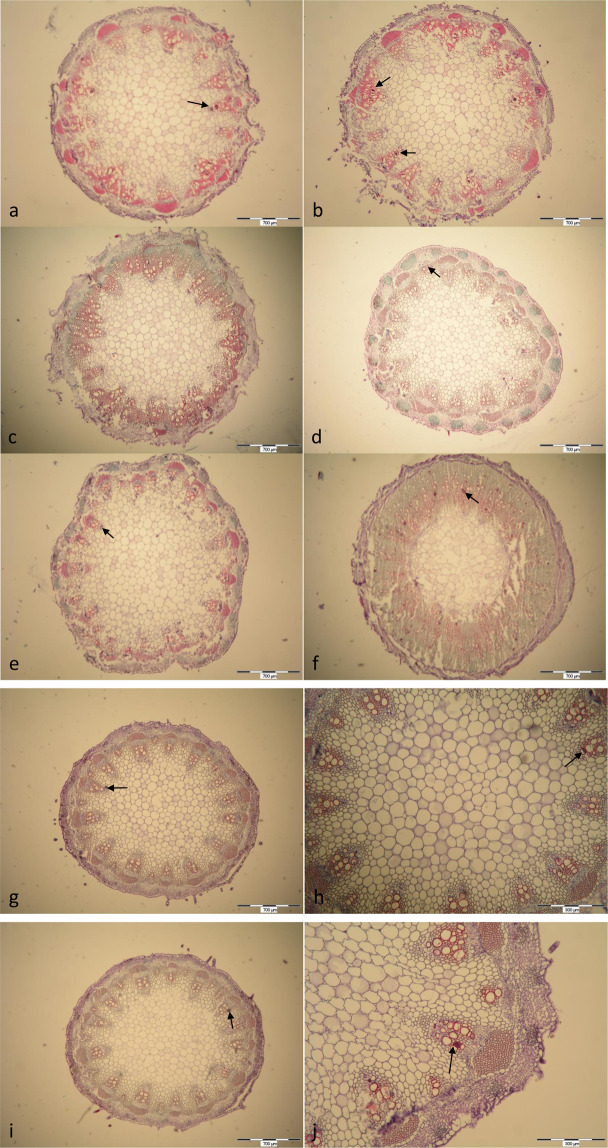
Cross sections of cut stems of garden cosmos: (**a**) – in water, full bloom; (**b**) – in water, loss of decorative value; (**c**) – in 8-HQC, full bloom; (**d**) – in 8-HQC, loss of decorative value; (**e**) – in 8-HQC + 2% (w/v) S, full bloom; (**f**) – in 8-HQC + 2% (w/v) S, loss of decorative value; (**g**) – in NS, full bloom; (**h**) – in NS, loss of decorative value; (**i**) - in NS + 2% (w/v) S, full bloom; (**j**) - in NS + 2% (w/v) S, loss of decorative value. ↖ - blocked vessels.

Both experimental factors, the day of observation and the treatment, significantly affected the number of vessel blockages (Table 2). No blocked vessels were observed in intact cosmos stems (data not presented); in cut stems kept in vase solutions the first blockages appeared on Day 2 after harvest. In stems held in water, up to 14.5% of vessels were already completely or partially blocked (Table 2, Fig. 1a). In stems held in the biocide solutions on Day 2 practically all vessels were clear (0 and 0.5% of blocked vessels in 8-HQC and NS respectively, Fig. 1c,g). Sucrose in the biocide solutions appeared to enhance blockage formation: on Day 2, ca. 6% of vessels were blocked in 8-HQC with sucrose and 5.5% in NS with sucrose (Table 2, Fig. 1e,i). On Day 4, the number of blockages increased in all solutions (Table 2, Fig. 1). In stems kept in water ca. 16.5% of vessels were completely or partially blocked (Table 2, Fig. 1b). The highest frequency of blockages (36.5%) was observed in stems held in the standard preservative – more than twice as many as in the control treatment and nine times more than in pure 8-HQC (Table 2, Fig. 1d,f). The vessels were completely blocked. Ca. 14.5% of completely or partly blocked vessels were observed in stems kept in NS with sucrose, i.e. twice as many as in stems held in pure NS solution (7%, Fig. 1h,j).

**Table 2:** Proportions of blockages in the xylem of cut cosmos stems (means of 3 observations of cross sections from 3 stems).

Treatment	Blocked vessels (%)	
	Full bloom Day 2 after harvest	Initial wilting Day 4 after harvest
H_2_O (control)	14.5 ± 0.8 d^a^	16.5 ± 0.8 d
8-HQC	0.0 ± 0.0 a	4.0 ± 0.2 b
8-HQC + 2% (w/v) S	6.0 ± 0.3 b	36.5 ± 1.8 e
NS	0.5 ± 0.0 a	7.0 ± 0.4 c
NS + 2% (w/v) S	5.5 ± 0.3 b	14.5 ± 0.7 d

^a^ the same as in Table 1.

The relative water contents in ray florets varied depending on the treatment and the day of measurement (Table 3). No significant changes in the RWC were observed in intact flowers during 6 days of the experiment. In control cut flowers, the RWC remained stable for the first 4 days and only on Day 6 a dramatic drop in the water content was observed. On Day 2, the RWC in flowers from all holding solutions was lower than in intact flowers or those kept in water. On Day 4, flowers held in both NS solutions had a lower RWC than those from the 8-HQC solutions or water. On Day 6, the highest RWC values were observed in intact flowers and those held in pure NS solution, and they did not differ significantly from the initial level. The lowest amounts of water on Day 6 were observed in flowers held in water and 8-HQC. Flowers held in the mixtures of the two biocides with sugar had approximately half of the water content of that on the day of harvest.

**Table 3:** The relative water content (RWC) in ray florets of cut garden cosmos.

Treatment		RWC (%)		
		Full bloom Day 2 after harvest	Initial wilting Day 4 after harvest	Total loss of decorative value Day 6 after harvest
Intact flowers		73 ± 2.3 efgh^a^	70 ± 4.1 efg	64 ± 6.7 def
Cut flowers	H_2_O (control)	75 ± 6.7 fgh	79 ± 3.4 gh	21 ± 5.1 a
	8-HQC	60 ± 7.1 de	81 ± 5.9 h	29 ± 4.7 ab
	8-HQC + 2% (w/v) S	64 ± 8.5 def	81 ± 3.1 h	36 ± 3.6 bc
	NS	68 ± 5.2 def	70 ± 6.2 efg	69 ± 6.1 defg
	NS + 2% (w/v) S	58 ± 7.5 d	66 ± 8.7 def	39 ± 4.7 bc

^a^explanation as in Table 1. Immediately after harvest the RWC was 73%.

Both, the vase solution and the analysis day significantly affected the total glucose contents in ray florets (Table 4). The ray florets, whether cut or left on mother plants, were losing carbohydrates during aging but at different rates. After a dramatic drop in the sugar level on Day 4, on Day 6 the intact flowers had still two thirds of the initial sugar content. Cut flowers held in water and in the 8-HQC solution lost over 75% of their glucose  during vase life while those in the NS solution lost about 50%. When sucrose was present in the holding solution, glucose  losses were smaller: ca. 30% in the standard preservative and only 7% in the mixture of NS with sucrose. At the end of the vase life, flowers from the NS + sucrose treatment had 40% more sugars than the intact flowers, and four times more than those held in water.

**Table 4:** Total glucose contents in ray florets of cut garden cosmos.

Treatment		Total glucose (mg ·g^−1^DW)		
		Full bloom Day 2 after harvest	Initial wilting Day 4 after harvest	Total loss of decorative value Day 6 after harvest
Intact flowers		556.0 ± 31.4 h^a^	114.7 ± 20.0 a	368.1 ± 34.7 fg
Cut flowers	H_2_O (control)	285.1 ± 36.2 cde	210.7 ± 3.5 b	130.2 ± 11.8 a
	8-HQC	286.8 ± 21.1 cde	134.0 ± 12.7 a	105.6 ± 17.6 a
	8-HQC + 2% (w/v) S	399.4 ± 14.2 g	303.8 ± 15.8 de	399.1 ± 55.3 g
	NS	249.3 ± 7.6 bc	249.0 ± 10.4 bc	267.6 ± 6.8 bc
	NS + 2% (w/v) S	297.8 ± 11.1 de	231.3 ± 19.0 bc	516.1 ± 16.3 h

^a^the same as in Table 1. Immediately after harvest total glucose content was 556.0 mg g^−1^ DW.

After the initial drop on Day 2, the MDA content increased in most cases. In intact flowers the MDA levels were higher than in flowers from any of the holding solutions (Table 5). The lowest MDA amount was observed in flowers held in the 8-HQC solution, significantly less than those kept in the NS solution. Supplementing the biocides with sucrose resulted in an increase in MDA in flowers.

**Table 5:** The malondialdehyde (MDA) content in ray florets of garden cosmos.

Treatment		MDA (nmol·g^−1^DW)		
		Full bloom Day 2 after harvest	Initial wilting Day 4 after harvest	Total loss of decorative value Day 6 after harvest
Intact flowers		3.2 ± 0.1 a^a^	5.7 ± 1.1 ef	8.3 ± 0.3 i
Cut flowers	H_2_O (control)	4.5 ± 0.4 bcd	6.2 ± 0.3 fg	7.0 ± 0.7 h
	8-HQC	5.0 ± 0.4 de	6.2 ± 0.5 fg	4.0 ± 0.4 bc
	8-HQC + 2% (w/v) S	3.9 ± 0.2 ab	4.9 ± 0.3 d	6.3 ± 0.4 fgh
	NS	4.7 ± 0.2 cd	4.6 ± 0.2 bcd	5.3 ± 0.5 de
	NS + 2% (w/v) S	3.9 ± 0.1 ab	5.2 ± 0.1 de	6.1 ± 0.1 fg

^a^the same as in Table 1. Immediately after harvest the MDA content was 5.5 nmol g^−1^ DW.

Significant differences were observed in the hydrogen peroxide contents in the ray florets (Table 6). In intact flowers, there was a threefold increase in its amount between Day 0 and Day 6, while in cut flowers held in water it fell to one fourth of that in intact flowers. The lowest levels of H_2_O_2_ were found in flowers from both 8-HQC solutions – without and with sucrose. In flowers kept in the NS-containing holding solutions, after initial reduction on Day 2, the amount of H_2_O_2_ increased and on Day 6 it was 2–3 times higher than in flowers from 8-HQC and 8-HQC + S, respectively.

**Table 6:** The hydrogen peroxide content in ray florets of garden cosmos.

Treatment		Hydrogen peroxide (µg·g^−1^DW)		
		Full bloom Day 2 after harvest	Initial wilting Day 4 after harvest	Total loss of decorative value Day 6 after harvest
Intact flowers		1115.6 ± 127.5 fg^a^	2270.1 ± 60.0 j	3551.2 ± 238.2 k
Cut flowers	H_2_O (control)	1116.3 ± 124.4 fg	1272.5 ± 101.3 gh	847.0 ± 107.4 cd
	8-HQC	1069.9 ± 80.8 ef	844.1 ± 80.0 cd	559.8 ± 41.4 a
	8-HQC + 2% (w/v) S	934.9 ± 62.1 de	1213.4 ± 7.6 gh	648.0 ± 118.7 b
	NS	847.4 ± 10.6 cd	1365.9 ± 67.6 h	1119.3 ± 128.4 fg
	NS + 2% (w/v) S	750.3 ± 58.3 bc	1195.2 ± 38.9 fg	1922.4 ± 97.8 i

^a^the same as in Table 1. Immediately after harvest the hydrogen peroxide content was 1115.6 µg g^−1^DW.

Due to an interaction of the treatment and the analysis day, ray florets differed significantly in catalase activity (Table 7). On Day 2, the catalase activity was elevated in cut flowers from all treatments but this increase was the greatest in flowers held in water – over 6-fold relative to Day 0 and to that in intact flowers on Day 2. Generally, on Day 4 the catalase activity dropped relative to Day 2. On Day 6, it increased in intact flowers – 2.7 times more as compared to the initial value, and it was the highest on that date. The lowest activity was in flowers held in solutions of both biocides; the sucrose supplement significantly increased the catalase activity, comparable to that in control flowers held in water.

**Table 7:** The catalase activity in ray florets of garden cosmos.

Treatment		Catalase activity (mkat·g^−1^DW)		
		Full bloom Day 2 after harvest	Initial wilting Day 4 after harvest	Total loss of decorative value Day 6 after harvest
Intact flowers		733.8 ± 28.6 bc^a^	441.3 ± 68.0 a	1975.0 ± 349.9 e
Cut flowers	H_2_O (control)	4513.5 ± 912.9 g	1731.3 ± 131.6 de	867.9 ± 72.3 bc
	8-HQC	2240.5 ± 522.2 e	1448.8 ± 178.6 de	286.6 ± 98.1 a
	8-HQC + 2% (w/v) S	3189.3 ± 140.1 f	582.5 ± 78.9 a	721.5 ± 32.7 bc
	NS	1846.3 ± 282.2 de	1506.6 ± 244.5 de	552.1 ± 67.5 a
	NS + 2% (w/v) S	1750.1 ± 255.8 de	1822.0 ± 50.4 de	1116.6 ± 47.6 cd

^a^the same as in Table 1. Immediately after harvest the catalase activity was 733.8 mkat g^−1^DW.

**Figure 2:**
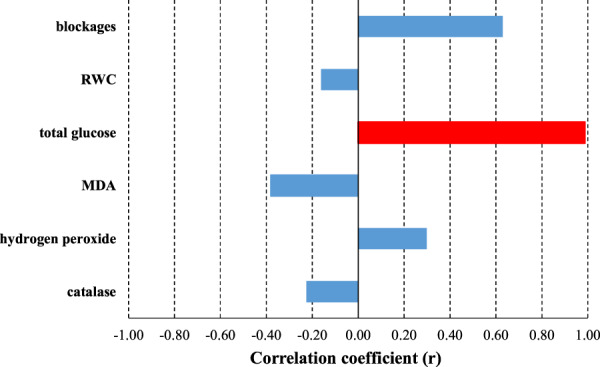
Correlation coefficient values for the relationships of vase life of cut garden cosmos flowers placed into holding solutions and senescence parameters. Data were subjected to Pearson correlation analysis. Vertical bars represent correlation coefficient values. Asterisks (**) represent significant differences at P < 0.05.

Correlation analysis was performed to determine the possible relationships between vase life of cut garden cosmos flowers placed into holding solutions and all senescence parameters measured. Most of these parameters were not significantly correlated with vase life (Fig. 2). Only the total glucose content was highly positively correlated with vase life of garden cosmos (r = 0.987; P = 0.002; Fig. 2).

## Discussion

The most commonly used “flower food” is composed of sucrose as an energy source and a biocide, usually 8-hydroxyquinilone citrate, to control bacterial growth. This is “the standard preservative”^9^. In this study, this standard preservative increased the longevity of cut cosmos flowers by ca. 70%. Sucrose was used here in the concentration of 2% (w/v); according to Redman *et al*.^2^, concentrations of 4% (w/v) and 8% (w/v) are ineffective in cosmos. The presence of sugar in the vase solution is indispensable as it provides a respiratory substrate and increases *osmoticum* thus improving water uptake. A biocide must be added simultaneously to vase water as in a pure sugar solution bacterial growth would be stimulated, limiting the water uptake and its transport in cut stems. Continuous water influx into flowers is crucial for their opening and their further life^24^.

The 8-hydroxyquinilone citrate is a widely tested and used biocide for sugar-containing holding solutions for cut flowers. Another such biocide, relatively new on the market, is nanosilver. It is perceived to be a potent antimicrobial agent^10^ with fairly limited data on its action and effects. It was chosen here for comparisons with the commonly used 8-HQC. The mechanism of action of silver in nanoparticles is not completely understood and only partial explanations of its mode of action in cut flowers have been published^17,18^. There are numerous reports that it prolongs the flower vase life^7,14–18^. Here, in cosmos, it also increased flower longevity, by as much as 50% relative to water, but its effect was not statistically different from that of the 8-HQC.

Observations made in this study suggest that silver nanoparticles may be more potent than 8-HQC in limiting the formation of xylem blockages. Both tested biocides in pure water solutions were comparably effective and the vessels remained practically free of blockages on Day 4. For sucrose solutions, NS was significantly better than 8-HQC in limiting stem blockages: there were 2.5 time fewer of those relative to the standard preservative (8-HQC + sucrose).

The nature of blockages in cosmos stems was not studied; however, bacteria are the most common cause of obstruction in water uptake by cut flowers^4^. It appears plausible that NS limits bacteria growth in xylem^7,17,18^. Unfortunately, a reduction in stem blockages did not translate in this case into improved flower longevity. Actually, the longevity of stems held in the sugar-containing solutions was somewhat better, despite a higher proportion of blocked vessels. This is in line with de Stigter’s opinion that “the phenomenon of rapidly declining overall water uptake of cut roses is not caused by a massive, solid plugging of the xylem vessels, as almost generally inferred in the literature”^3^. A somewhat similar situation was observed in cut clematis flowers where the postharvest longevity was not associated with the vessel size, and the vessel size would appear decisive for the ability to conduct water^25^. This suggests a need for a closer look at the relationship between water balance and the longevity of cut flowers.

The water balance in cut flowers can also be evaluated by the relative water content of petals. The RWC in intact cosmos flowers was higher than that in cut flowers. That was expected, as flowers still on the mother plant are not subjected to the water stress created by harvest. In cut cosmos, on Day 6 after harvest, i.e. the last date of observations, the RWC with NS present was higher than that with 8-HQC, but only when stems were held in pure biocide solutions. Addition of sucrose produced significant reductions in the water content of flowers, regardless of the biocide used. Again, a discrepancy occurred between the positive effects of biocides on cosmos longevity and a diversified response to their presence of the water-related parameter. In roses, pulse treatments with NS extended the flower vase life in association with a relatively high RWC of leaves, perhaps because of a reduction of the stomata aperture^26,27^. Cut cosmos stems are devoid of foliage implying that there must be another explanation for a better effect of NS.

The concentration of soluble sugars is important for water influx into petals. In senescing flowers – both intact and cut - the endogenous sugars are depleted^9^. Those present in the flower preservatives are up taken and metabolized, extending life processes hence contributing to a cut flower longevity. These soluble sugars often accumulate in petals where they serve as *osmoticum* improving water influx. In this study, the levels of total glucose in intact flowers dropped by 1/3 during the six days of experiment, while in control cut flowers that drop amounted to 3/4 of the initial level. This can be considered normal, as senescence is hastened by detaching a flower from a mother plant^3–6^. A similar reduction in soluble sugars in ray florets of cosmos was also reported by Jani and Mankad^28^. Here, both biocides mitigated the total glucose reduction and NS appeared more efficient than 8-HQC, both in pure solution and in the presence of sucrose.

To maintain full turgidity flower cells have to maintain their membranes intact. During senescence these membranes undergo peroxidation. Van Doorn *et al*.^29^ in their studies on *Iris* showed that in senescing flowers, cell membrane components degenerate and are remobilized. One of the degradation products is MDA and this can be readily measured, providing an indirect indicator of the membrane condition. Here, the MDA contents kept increasing in all flowers, including the intact ones, where the MDA level was the highest after six days. Similarly increasing MDA levels were also observed in senescing cut carnations^30^, intact *Coreopsis* flowers as well as in cut marigolds^31^ and daylily^32^. An unexpected phenomenon in cosmos was the high MDA level in flowers held in the preservatives which were supposed to maintain membrane properties. The lower MDA contents, thus a better membrane condition than in control flowers, were observed in flowers held in pure biocide solutions and here 8-HQC seemed slightly more effective than NS.

Senescence and a loss of the decorative value of flowers are associated with the generation of the reactive oxygen forms (ROS). One of them is the hydrogen peroxide which – depending on its concentration – can induce an oxidative stress or serve as a signal transmitter. Hossain *et al*.^21^ reported an increase in H_2_O_2_ in senescing gladiolus flowers with its peak level in completely faded florets. Here, the highest H_2_O_2_ level was observed in intact flowers: after six days its amount was several times higher than that in cut flowers. This contrasts with the observations of Arrom and Munné-Bosch^33^ on lilies, where an increase in ROS levels was far greater in detached flowers than in flowers remaining on the plant. Those authors suggested that the ROS increases may be much more strongly associated with petal senescence in cut flowers than in naturally senescing petals, exactly the opposite to what was observed here. Here, only the 8-HQC solution significantly reduced the accumulation of hydrogen peroxide in senescing cosmos florets. Pure NS and both preservatives were not effective in this regard.

Studies on the effects of flower preservatives on the activity of the antioxidative enzymes are scarce. CAT performs a key role in detoxification of H_2_O_2_^20^. According to Chakrabarty *et al*.^23^, the activity of catalase in cut chrysanthemums kept increasing until full bloom and later fell dramatically. In gladiolus, the fluctuations in the catalase activity were observed during flowers senescence^34^. In intact cosmos flowers, the activity tripled in six days of the experiment while in cut control flowers it dropped, similarly to flowers held in both 8-HQC solutions. In flowers placed into NS + sucrose, the catalase activity was higher than that in flowers from all other holding solutions, or at the day of harvest. Evidently, the presence of sucrose in the NS solution stimulated the enzyme activity in flowers. This may be an indication of their greater ability to eliminate hydrogen peroxide. However, the presence of H_2_O_2_ and the catalase activity in both intact and cut flowers allows us to speculate that all of them were under some sort of stress and that detaching stems from mother plants was not the only stimulus inducing it. As for the oxidative stress, differences in the flower response to 8-HQC and NS were evident but they do not explain the mechanism of action of NS.

In studies on cut spray carnations^18^ it was speculated that the positive effects of NS may be partially attributed to its anti-ethylene action. Ag+ ions released from NS effectively block the ethylene effects on flowers. The senescence in the flowers of Asteraceae is not stimulated by ethylene^35^. However, both in carnations^17,18^ and in garden cosmos, NS acts synergistically with sucrose in extending cut flower longevity.

In conclusion, while the incidence of xylem blockage in cosmos could be modified by holding solutions, generally, their effects on vascular occlusions and on changes in RWC as well as on several senescence-related processes were not directly associated with the postharvest longevity of cosmos. Solutions containing nanosilver gave comparable results to those of 8-HQC so NS may be recommended as a component of a flower food for cut cosmos flowers.

## Material and Methods

Plants of *Cosmos bipinnatus* ‘Sonata Carmine’ were grown in the fields of the Department of Ornamental Plants of the Warsaw University of Life Sciences. Stems were harvested at the stage of open flower heads, with no visible defects such as mechanical injury, or symptoms of disease or pests. They were immediately transferred to a laboratory, trimmed to 40 cm and placed in several solutions: distilled water, solutions of 8-hydroxyquinoline citrate (8-HQC) or nanosilver (NS) in distilled water, the standard preservative composed of 200 mg·dm^−3^ 8-HQC plus 2% (w/v) sucrose (8-HQC + 2% (w/v) S), or a mixture of 5 mg·dm^−3^ solution of nanosilver with 2% (w/v) sucrose (NS + 2% (w/v) S). The vases with flowers were placed in a phytotrone under controlled conditions: relative humidity (RH) 60%, 20 °C, 12 h light from fluorescent lamps of 25 μmol·m^−2^·s^−1^. For the vase life evaluation, 10 flowers individually tagged and treated as single replications were used. Separate vases with flowers for analyses were prepared as above. The vase life was considered terminated when symptoms such as wilting or fading occurred on the ray florets.

To determine the frequency of xylem blockages in stems from the holding solutions, stem base fragments 0.5 cm in length were sampled at the stage of full bloom (Day 2 after harvest) and on Day 4 when the initial wilting symptoms occurred (3 samples per treatment). The plant material was fixed according to Hause *et al*.^36^ in the PFA fixative: 4% paraformaldehyde (Sigma), 0.4% DMSO (Sigma), 0.05 M phosphate-buffered saline (PBS) (pH 7.0), DEPC-treated water (Sigma) for 12 h under 0.6 atm. Fixed samples were washed twice for 30 min. each in the phosphate-buffere saline (PBS), dehydrated in the graded ethanol series (30, 50, 70, 80, 95, 100%), each series for 1 h in RT (room temperature), and twice in Histoclear (Histochoice Clearing Agent, Sigma) for 30 min each. Paraplast pellets (Sigma) were added to the last series of Histoclear in the paraffin oven, twice a day for 5 days, at 58 °C, until the Histoclear evaporated completely. In the last step, specimens were embedded in clear Paraplast (Sigma) and sectioned at 10 µm thickness on a rotary microtome (Leica RM 2255). All preparations were made on the RNase, DNase-free slides (Thermo ScientificMenzelGläser, Superfrost Plus), and dried at 42 °C for 2–4 days. For identification of xylem occlusions in stems, permanent slides were stained using the Kartel’s quadruple stain (safranin – crystal violet – fast green – orange G). The specimen were observed under brightfield microscope (Olympus BX 41) and the microphotographs were taken with digital camera Olympus C-5050 and the software Quick Photo Pro. Vessel blockage was expressed as a percentage of blocked vessels in the total number of vessels on a cross section of a stem.

The relative water content (RWC) was measured in 10 samples in each treatment. Ray florets were weighed, placed on paper in a Petri dish and covered with distilled water for 24 hours, then dried, weighed while fully turgid and placed in an oven at 105 °C for 72 hours. The dry matter was weighed twice – immediately after taking out of the oven and after further 30 min drying. No difference in weight between these two measurements indicated complete drying. The RWC was measured in three phases of the flower head development in both, cut and intact flowers: in full bloom (Day 2), when the initial wilting symptoms appeared (Day 4) and at the stage of a total loss of decorative value (Day 6). On the same dates the material was sampled from intact plants in the field. The values were calculated according to the formula of Smart and Bingham^37^ and given in %.

$${\rm{R}}{\rm{W}}{\rm{C}}\,=\,\frac{{\rm{f}}{\rm{r}}{\rm{e}}{\rm{s}}{\rm{h}}\,{\rm{w}}{\rm{e}}{\rm{i}}{\rm{g}}{\rm{h}}{\rm{t}}-{\rm{d}}{\rm{r}}{\rm{y}}\,{\rm{w}}{\rm{e}}{\rm{i}}{\rm{g}}{\rm{h}}{\rm{t}}}{{\rm{f}}{\rm{u}}{\rm{l}}{\rm{l}}{\rm{y}}\,{\rm{t}}{\rm{u}}{\rm{r}}{\rm{g}}{\rm{i}}{\rm{d}}\,{\rm{w}}{\rm{e}}{\rm{i}}{\rm{g}}{\rm{h}}{\rm{t}}-{\rm{d}}{\rm{r}}{\rm{y}}\,{\rm{w}}{\rm{e}}{\rm{i}}{\rm{g}}{\rm{h}}{\rm{t}}}\times 100$$

Biochemical analyses were done on Day 0, Day 2, Day 4 and Day 6 after harvest. Florets of 30 flower heads from each treatment were finely chopped, mixed, and samples of 0.5 g were taken. They were stored at −80 °C until analyzed. Three analyses of each of 3 samples (extracts) were done for each data point.

Total glucose were extracted in hot 80% ethanol and measured according to Dubois *et al*.^38^. The sugar content was calculated according to the standard curve made for glucose and expressed as mg·g^−1^ DW. Malondialdehyde was measured according to Hodges *et al*.^39^, based on the color reaction with the thiobarbituric acid and its values were given in nmol·g^−1^ DW. The hydrogen peroxide levels were measured according to Siedlecka^40^ and given in µg·g^−1^ DW. The catalase activity (CAT, EC 1.11.1.6) was measured according to Goth^41^ and given as mkat·g^−1^DW.

The results of analyses were subjected to a single- or two-factorial analysis of variance ANOVA using the SPSS Statistics 21.0 program. The significance of differences between the means was evaluated by the Duncan’s test at P = 95%. To clarify the correlations between vase life of cut garden cosmos flowers placed into holding solutions and senescence parameters, Pearson correlation analysis was performed.
